# The extent to which the design of available reproductive health interventions fit the reproductive health needs of adolescents living in urban poor settings of Kisenyi, Kampala, Uganda

**DOI:** 10.1186/s12889-021-10933-3

**Published:** 2021-05-17

**Authors:** Doreen Tuhebwe, Susan Babirye, Steven Ssendagire, Freddie Ssengooba

**Affiliations:** Department of Health Policy, Planning and Management, School of Public Health, Makerere University, Kampala, Uganda

**Keywords:** Adolescents, Reproductive health, Interventions, Adolescent needs, Slums, Uganda

## Abstract

**Background:**

The rate at which informal urban settlements (slums) are developing in Low and Middle Income. Countries (LMICs) like Uganda is high. With this, comes the growing intersection between urbanization and the reproductive health of key populations. Currently, a number of interventions are being implemented to improve the Reproductive Health (RH) of adolescents in Kisenyi, the largest informal urban settlement in Kampala, the capital of Uganda. Despite these efforts, adolescent RH indicators have persistently remained poor in Kisenyi. This could be indicative of a gap between the provided and needed adolescent RH interventions. We assessed the fit between the available interventions and the RH needs of adolescents living in Kisenyi.

**Methods:**

We conducted a qualitative study in July 2019–February 2020 in Kisenyi. The methodology was guided by the Word Health Organization global standards for quality-health care services for adolescents, the “For whom? Where? By whom? and What?” Framework of sexual RH service delivery and the realist evaluation approach. Eight focus group discussions were conducted with adolescents 15–19 years to explore their RH needs. The design and implementation of the available adolescent RH interventions were assessed by conducting Key Informant interviews with 10 RH service providers in Kisenyi. Validation meetings were held with adolescents and they scored the extent to which the various design features of the existing interventions fit the adolescents’ RH needs.

**Results:**

The available RH interventions focused on meeting the sexual RH needs like providing family planning services but less on social needs like livelihood and sanitation which the adolescents identified as equally important. While the providers designed intervention to target 10-24 year olds, the adolescents preferred to have interventions that specifically targeted the study population 15-19 years. Most interventions were facility-based while, the adolescents desired community based outreaches.

**Conclusion:**

The packaging and mode of delivery of interventions were perceived less holistic to meet the adolescents’ needs. Most interventions were designed to address the sexual and family planning needs while ignoring the wider social and livelihood needs. More holistic and outreach-based programming that addresses RH within the broader context of livelihood and sanitation requirements are more likely to be effective.

## Background

As countries work towards attainment of health and wellbeing for all (Sustainable Development Goal 3), adolescents especially those living in informal settlements are being left behind. This is evidenced by their persistent poor health indicators of HIV prevalence and adolescent pregnancy [1, 2] especially in low middle-income countries (LMICs). These indicators are worse among adolescents living in poverty stricken settings [2].

For example, while the HIV prevalence among Ugandan adolescents ages 15–19 is 2.4%, the prevalence of HIV among sexually active youth living in the slums of Kampala was reported at 13.9%-much higher than the national prevalence [3, 4]. According to the 2016 Uganda Demographic Health Survey, 16.8% of adolescents aged 15–19 in the Capital Kampala had begun child bearing and this is believed to be worse in urban poor set ups like Kisenyi (the largest slum in Kampala). This is due to compounded vulnerabilities faced by adolescents such as low education and poverty [5] and lack of access to appropriate reproductive health interventions for this vulnerable population [6]. Studies have also shown poorer intermediate outcomes like age at first sex, age at first child birth and age at first marriage occurring earlier for the poorer adolescents than those from the richest wealth quintile [2]. With 27% of adolescents aged 13–17 years being sexually active in Kampala [7], reproductive health for the urban poor is an urgent priority in order to meet the 2030 SDG targets related to sexual and reproductive health and rights for all adolescents [8].

Although slums are in urban areas where service delivery points are commonly available, this geographic proximity does not facilitate easier access to health services [9, 10]. Other access challenges such as financial barriers impede access to health care services by slum dwellers in Kampala [11, 12]. Studies on the health status of the urban dwellers demonstrate inequities in health indicators between the general population and the slum dwellers [13]. With the increasing population growth rate in Africa [14] and Uganda in particular, the reproductive health of the urban poor is of importance. The lives of adolescents living in the slums of Kampala are complicated by many additional adversities due to the living conditions. These exacerbate their vulnerability such as; their dependency, inexperience, lack of positive guidance [15], food scarcity, abuse and neglect [16]. These result into reproductive health problems such as early unwanted pregnancy, unsafe abortion, STls/HIV/AIDS, psycho-social problems such as substance abuse, delinquency, truancy and sexual abuse. These adolescents are in need of adequate RH interventions.

In Uganda, the current policy environment enables adolescent health programming [17]. Young people have been recognized as a critical national resource and their health as a worthwhile investment for future growth and development [15]. In order to improve teenage and adolescent RH outcomes in the urban poor population of Kisenyi slum, public institutions, NGOs and implementing partners in Uganda have implemented several reproductive health (RH) interventions especially youth friendly corners at public health facilities to avail services such as youth counseling, access to family planning and HIV/AIDS services [18]. Adolescents also need education and social services in order to comprehensively fulfil their livelihood and development needs. Providers and systems need to be geared towards meeting the priority needs of the adolescents [19] but many time the design and programming of these services are less satisfactory to the adolescents especially in the urban poor settings [10, 20]. This is indicative of a gap between the provided and needed RH interventions.

Several studies have proposed “what works” for evidence-based adolescent health programs [21]. Depending on the design and implementation of the intervention, adolescent interventions can be broadly divided into behavioural interventions, which seek to change the knowledge, skills and attitudes of individuals and structural interventions, which aim to tackle broader societal issues that drive the spread of STIs [22] and the two approaches are not mutually exclusive. The “For whom? Where? By whom? and What?” Framework of sexual reproductive health service (SRHS) delivery by Denno and colleagues [6] can guide the assessment of the design of existing adolescent RH interventions. Examples of RH intervention design considerations include: whether the intervention is school or community based (context); whether the intervention targets one or both males and females, and consideration of intended intervention outcomes - for example, delayed initiation of sex, prevention of infection (HIV/ STIs) or reduced number of sex partners. Such design considerations can be used to assess whether interventions are fit for a given population or context.

Literature shows that during adolescence, an individual acquires the physical, cognitive, emotional, social, and economic resources that are the foundation for later life health and wellbeing [23]. Therefore, in adolescent interventions, there is need to tackle preventable and treatable adolescent health problems including infectious diseases, under nutrition, HIV, sexual and reproductive health, injury, violence and mental health including substance abuse management. Standard 3 of the WHO global standards for quality health-care services for adolescents provides a framework that can be used to view adolescent needs in a comprehensive way [24]. Standard 3 focusses on providing “appropriate package of services” which can meet medical, social and developmental needs of the adolescents. This WHO framework speaks to complementary actions needed to promote healthy development in adolescents and gives direction for the need of interventions to decrease and mitigate adolescent vulnerability [25].

In order to inform efforts to improve the design and implementation of adolescent RH interventions in urban poor setting in Uganda and beyond, this study addressed three research questions. 1) What are the RH needs of adolescents 15–19 years living in the urban poor settings of Kisenyi? 2) What is the design of the available RH interventions in the study area and 3) To what extent is the design of the available RH interventions aligned with the RH needs of these adolescents?

## Methods

### Study site

This study was conducted in Kisenyi Slum located in Kampala city, Uganda. Kampala city has the highest urban population growth rate in Uganda [26] and Kisenyi slum is the largest slum/ informal settlement located in the south-western part of Kampala Central Division [27]. Kisenyi has typical urban poor characteristics of; 1) informal settlements, 2) clusters of dilapidated housing, 3) large population size and 4) a rapidly expanding population [28]. Kisenyi is also categorized as a slum according to the urban planning documentation from Kampala Capital City Authority. Kisenyi is comprised of 3 parishes: Kisenyi I, Kisenyi II and Kisenyi III. Kisenyi II Parish is residential with a more stable population [29] and was purposively selected as the study area to give a representation of a typical adolescent that resides in this slum. Among the government aided adolescent RH investments in Kisenyi is a youth friendly corner at Kisenyi HCIV which has community outreaches through community health workers, also avails free condom dispensers in the community and radio sexual and RH programs for the youth [30]. Kisenyi HCIV also has affiliations with some institutions that majorly provide HIV/AIDS services. Other NGO led investments include youth health and sports centers and a few youth vocational training centers. Kisenyi has a public primary school under universal primary education and other social services like churches, sports play grounds, faith based home shelters and some privately owned group homes.

### Study design

This was a case study of a slum setting that employed mainly qualitative methods and was conducted between July 2019 and February 2020.

### Study population

The primary study population were adolescents aged 15–19 years living in Kisenyi slum. The study also included implementers of adolescent RH interventions as key informants.

### Data collection

To explore the Reproductive Health needs of the adolescents, eight focus group discussions (FGDs) were conducted involving 85 participants. The FGDs were held with the adolescents aged 15–19 years and had lived in Kisenyi for the last 1 year. The eligible adolescents were purposively identified by village health team (VHT) members-a cadre of community health workers. Once identified, the VHTs mobilized them to a central community venue decided upon by VHTs and the adolescents. At the venue, the researchers privately screened each adolescent by asking each one of them secretly (self-report) in order to stratify them by sex, schooling status, being sexually active, marital status and ever having a child i.e. criterion sampling [31]. Five FGDs were for males and three for females with details per sampling criteria in Table 1. Written informed consent was sought from the study participants aged 18–19 years, emancipated minors aged 15–17 years and from parents/guardian of adolescents below 18 years (for adolescents still under parental custody at the time the VHTs invited them to the venue). Written assent was sought from non-emancipated minors/adolescents aged 15-17 years.

**Table 1:** Characteristics of the FGD participants

FGD identifier	Number of participants	Sex	Schooling status	Sexual activity	Marital status	Ever had a child
FGD1	13	M	In school	Not active	Not married	no
FGD2	10	M	Out of school	Active	Married	no
FGD3	10	M	Out of school	Active	Not married	no
FGD4	10	M	Out of school	Active	Married	Has a child
FGD5	10	F	In school	Not active	Not married	no
FGD6	13	F	Out of school	Active	Married	Has a child
FGD7	9	F	Out of school	Active	Not married	no
FGD8	10	M	In school	Active	Not married	no

During the FGDs the adolescents described their RH needs, listed key providers of RH and related interventions in their community based on the FGD guide developed by the authors for the purpose of this study. When describing the RH needs, the adolescents were probed to enlist their comprehensive needs in the medical, social and developmental domains according to standard 3 of the WHO global standards framework [24]. According to the standard, examples of medical needs are: information, counselling, diagnostic, vaccination, treatment and care services that fulfils the SRH needs of all adolescents. Social needs may include parental support, housing and education while, developmental needs may include substance abuse control and prevention of violence and unintentional injuries. The FGDs were conducted in the local language known to all participants that is Luganda. Transcripts from the FGD were analyzed to summarize the list of medical, social and development needs mentioned by the Adolescents.

To describe scope and the design of available RH interventions, we conducted key informant interviews (KIIs) with service providers (urban authorities and implementing partners). We identified the first five KIs based on the discussion held with the adolescents when they listed key providers in Kisenyi. We identified the rest of the service providers using the snow balling technique by asking the key informants initially interviewed to name the other service providers that they know of who provide youth reproductive health interventions in Kisenyi. A total of 10 KIIs were conducted and their characteristics are shown in Table 3. The interviews were conducted using the KII guide developed by the authors for the purpose of this study. During the interviews, we first asked the service providers to describe the reproductive health interventions that they provide to the adolescents in Kisenyi. The “For whom? Where? By whom? and What?” Framework of SRHS delivery by Denno and colleagues [6] was adapted and used to interview service providers and urban authorities when asked to describe the scope of their interventions. For example, whether the intervention is school or community based or whether the intervention targets one or both males and females. We also probed the KIIs on whether they offered any services aligned with the medical, social and development needs that had been earlier mentioned by the Adolescents. In addition, we applied the principals of the realist evaluation [32] to probe the design and implementation features of the interventions that each provider was implementing in Kisenyi slum in order to assess whether they align with target population (adolescents’) context. The design features probed are summarized in Table 2.

**Table 2:** Intervention design features probed during Key informant interviews

Design feature	Definition of the feature
Scope of interventions	Probed the list of intervention that each provider is implementing according to the following categories: 1) health awareness (any intervention targeting to provide preventive and promotive health information); 2) violence prevention (any intervention to control violence, treat and rehabilitate victims); 3) mental health (any intervention for rehabilitation of psychosocial morbidities); 4) substance abuse (any intervention for drug use and tobacco use control); 5) sexual health (any intervention on safe sex counseling, family planning services access, HIV/AIDS testing and treatment and 6) maternal child health services (any intervention on safe motherhood and newborn care)
Target sub population	Probed the age group and sex being targeted by existing interventions
Delivery model used	Probed the level of implementation of the interventions e.g. whether facility based, school based, community based or policy based
Category of urban beneficiaries targeted	Probed whether interventions are designed to target urban poor or all urban beneficiaries irrespective of their context according to the realist evaluation approach
Mechanism of change envisioned	Probed whether mechanism of change for the intervention targets changing context in which adolescents live or influencing behaviour of the adolescents according to realist evaluation approach
Intended outcomes	Probed what the intended outcomes of the implemented interventions are for each intervention mentioned according to realist evaluation approach

Using content analysis, we summarized the number of providers implementing at least one intervention with the respective design features. Depending on the number of interventions provided within a respective design feature across the 10 service providers, each design feature was allocated counts to determine the most prevalent design characteristics. This information was visually presented using graphs and the qualitative quotes from the KIs provided the descriptions and explanations. Because of the broad definition of the design feature on “scope of interventions”, we presented the scope of this feature in a figure for better interpretation.

To determine the extent to which the available RH intervention designs fit the RH needs of the adolescents, we used deliberative multi-voting approach [33]. We held two community consultative validation meetings with 26 representatives of the adolescents engaged in the initial FGDs. This included 11 males in one validation meeting and 15 females in the other. During the meetings, 1) we presented to the adolescents the summary of their RH needs from the FGD analysis on a flip chart pasted on a wall and asked them to validate and prioritize the needs. The RH needs were categorized into sexual health and social needs as the main themes from the FGD analysis. Under each theme the adolescents were asked to individually choose their top 3 priority needs in sexual health category and top 2 priority needs in the social needs category. The adolescents used stickers to indicate their choice and each adolescent had a maximum of five stickers (three for sexual health and two for social needs).

2) The adolescents were also presented with the list of 10 service providers of RH interventions (named as provider 1, provider 2 and so on) with whom we held KIIs with. On the flip chart with the list of providers, we included a description of the nature of services per provider and the design features of each of the interventions provided. We asked the adolescents to score the extent to which the design of the interventions met their (adolescent) prioritized RH needs elicited in the sticker exercise described above. They scored the following design features that are adopted from the “For whom? Where? By whom? and What?” Framework of SRHS delivery, also described in Table 2 of the methods section: The key areas of inquiry were: 1) whether the intervention scope covered their priority RH needs; 2) whether the interventions targeted their age group; 3) whether the delivery model used for the interventions was adequate; 4) whether the interventions fit the urban poor context in terms of the beneficiary category and 5) whether the intended outcomes of the interventions aligned with their needs as articulated by them in the sticker exercise.

The scores were based on the scale of 0–5: 0 = not aware of the intervention or design used; 1 = Poor fit of the design; 2 = Moderate fit of the design; 3 = Good fit of the design; 4 = Excellent fit of the design and 5 = Exceptional fit of the design. A single score per design feature was collectively agreed by consensus in the respective male and female validation meetings and allocated for each provider (in the respective design feature of inquiry). After the meeting, the “male meeting” and “female meeting” scores were entered in excel and mean scores were derived for each design feature for all providers as a whole (aggregate score per design feature). During the meetings, the adolescents gave explanation for each score they agreed to allocate. After descriptive analysis, the aggregate mean scores were compared to the maximum possible mean score per design feature (which in this case was five) to determine the extent to which the design of the available RH interventions fit the RH needs of the adolescents. A radar chart was used to demonstrate the comparison. The radar chart plots the aggregate scores per design feature against the maximum possible mean score per design feature. The equi-angular spokes or radii show which of the design features is perceived to meet the adolescent RH needs the most and which one meets the adolescent needs the least across the interventions scored. The descriptive analysis was supplemented with the explanations provided by the adolescents during the validation meetings.

The FGDs, KIIs and validation meeting were facilitated by the Principal Investigators who have a good working knowledge of English and Luganda. During the interviews, open ended questions were asked followed by targeted questions (probes) on predetermined themes. Throughout the study, the team adhered to the RATS guidelines on qualitative research [34] in terms of 1) the Relevance of study design in answering the research question; 2) Appropriateness of qualitative method such as use of FGDs and KIIs; 3) Transparency of procedures in sampling respondents and 4) Soundness of interpretive approach as applied to the data analysis.

### Data management and analysis

During each interview, a Note taker was available to take notes. All the qualitative interviews were audio recorded with consent of the participants and then transcribed verbatim. The transcripts were analyzed using Atlas Ti vesion 8. A conventional content analysis approach was used for the qualitative data as described by [35]. The qualitative analysis was done in two stages; first, the manifest content analysis and then the latent content analysis. The transcripts were read and codes were derived by highlighting emerging issues based on our understanding of the data. Codes were then sorted into categories based on their linkages. The categories were grouped together into meaningful overarching themes i.e. RH needs of adolescents for objective one, design features of interventions for objective two and reasons for the assigned score on extent of fit of intervention design and RH needs for objective three. Key quotations that epitomized central themes related to the main findings are presented in the results. The Lead Researchers TD, BS and SS conducted the data analysis and synthesis. The information on the flip charts from the validation meeting scores was immediately entered into Microsoft excel to avoid loss of the hard copy data. Descriptive analysis was conducted and results presented in figures. Where appropriate, quantitative and qualitative results were presented together in order to enhance contextualization of the results.

### Ethical considerations

This study was approved by the Makerere University School of Public Health Higher Degrees Research and Ethics Committee (HDREC protocol number 669) and registered by the Uganda National Council of Science and Technology (Ref SS 5093). Permission was also sought from Kampala Capital City Authority and local leaders in Kisenyi Slum. The following ethical principles were upheld in the study; 1) anonymous data collection strategies to maintain a high level of confidentiality; 2) informants were asked to participate voluntarily; 3) written informed consent or assent; and 4) no study materials contained names or other explicit identifiers of participants. For confidentiality purposes, information on sexual undertakings of the adolescents discussed in the FGD were not attributed to any group member but was treated as generic needs for people in the age category. After each FGD, the study team demystified any misinformation, myths and misconceptions that were identified during the FGDs. All participants mobilized were given a modest fee of Uganda shillings five thousand (equivalent to USD 1.5) as refund for their transport to the venue. Permissions from participating organizations (service providers) was sought. Names of the participating organizations were obtained for use if corrective actions were to be undertaken and the organizations consented to have their names used in publication with the overriding principle of do no harm.

## Results

### Socio-demographic characteristics of study respondents

#### FGD participants

We conducted 8 FGDs with a total of 85 adolescents aged 15–19 years including 53 males and 32 females. Of these, 34/85 were currently in school, 23/85 were married and 51/85 were sexually active. In addition, 20/85 of the participants had ever had a child. During the data collection, we were not able to compose enough female participants for the FGDs of in school sexually active, and out of school sexually active married with no child.

#### Key informants

We interviewed 10 KIs. Table 3 below shows the KIs and the nature of services that they mainly provide.

**Table 3:** List of de-identified key informants and the nature of services that they mainly provide

KI Identifier	Nature of service provided
Provider1	Public Health Facility
Provider2	Cultural Based Reproductive Health Youth Center
Provider3	Urban Authority and Planners
Provider4	HIV/AIDS Provider for adolescent HIV testing and treatment
Provider5	HIV/AIDS Provider for most at risk populations
Provider6	Local Church
Provider7	Vocational Institute
Provider8	Community Health Workers
Provider9	Peer Educators
Provider10	Community Elders

#### Adolescents’ reproductive health needs

Two themes - sexual reproductive health and social needs, emerged from the qualitative analysis of the needs of adolescents.

#### Sexual reproductive health needs

Across all the FGDs the adolescents mentioned needs that pertain to sexual reproductive health. These needs were broadly about need for: sexual health information, RH services and products and service delivery arrangement with an emphasis on quality and adequacy. The needs under information and RH services and products included the need for: regular sexual health information, quality family planning services, quality STI/HIV/AIDS testing and treatment, adequate supply of quality condoms, antenatal care for young mothers, abortion services and newborn care services. During the validation meetings, the top three prioritized sexual RH needs for the females were quality family planning, quality STI/HIV/AIDS testing and treatment and accessing affordable (free) general medical services (in that order). The males’ priority sexual health needs were quality STI/HIV/AIDS testing and treatment followed by sexual health information.

*“As you know, we the youth may want to have sex and need protection … but sometime they [condoms] are faulty …*. W*hat we want is that they put for us dispenser boxes for good condoms in the community like provider 2 and 5 have been doing … the dispensers are always empty so you end up going to buy at the shop yet money is scarce and majority of us don’t have jobs* (Male participant FGD3).

“*… the testing exercise always frightens us. For example, if you are going to test for HIV, people start talking about you saying that “you are now sexually active”* (Female participant FGD5).

The needs around service delivery arrangements included need for: affordable (free) general medical services, youth friendly health workers (with a positive attitude), less waiting time at the public health facility and consistent holistic community health outreaches.

“*We have health centres here in Kisenyi, but most of them are private clinics and very expensive. When you go to the public facility, they make you walk around up to three or five times before getting the services you are looking for”* (Female participant FGD7).

#### Social needs

While we sought to explore adolescent RH needs, when describing their needs, the adolescents highly emphasized social needs in all the FGDs. These included need for: livelihood and decent employment, sanitation, parental guidance, substance abuse management in the community, housing and drug abuse rehabilitation. In the validation meetings, female participants prioritized livelihood and sanitation while, the male adolescents prioritized livelihood and housing in the order mentioned.

*“Jobs!...give us jobs that will build us …. When we have nothing, we end up stealing”*(Male participants FGD4).

“*The poverty is very high and when you get infected with diseases like Candida you can’t even go for treatment because of lack of money … ..We fall sick all of the time, the trenches here spread diseases due to poor sanitation”(*Female participant FGD7).

### Design features of the available adolescent RH interventions in Kisenyi slum

#### Scope of available RH interventions

During the KIIs, when the Key informants were asked to describe the reproductive health interventions that they provide to the adolescents in Kisenyi and the scope of the interventions, upon content analysis, it was noted that the service providers provide several interventions across the themes of health awareness, violence prevention, mental health, substance abuse, sexual health, maternal health and social welfare at varying degrees. For health awareness, 6/10 providers reported having at least one intervention with this focus. Sexual health had the biggest focus with 7/10 providers reporting that they provide at least one intervention with a sexual health focus.

*“At the public health facility we treat the adolescents for STDs, HIV...some of them have suffered from gender based violence, so we manage the injuries. (Provider 1).*

Social welfare was a new theme identified in our study. This theme covered livelihood, sanitation, housing and parental guidance and it had the least number of providers. For example, only 3/10 providers indicated that they implement at least one intervention with a focus on livelihood which included providers availing job creation training opportunities for the adolescents, providing seed funding and other material that can aid adolescents to have startups for work. Housing included initiatives where service providers group homes or shelters where homeless adolescents could live or spend the night while, parental guidance covered activities where providers put up initiatives to reach out to adolescents and gave them guidance and direction and they transition in life.

**Fig. 1:**
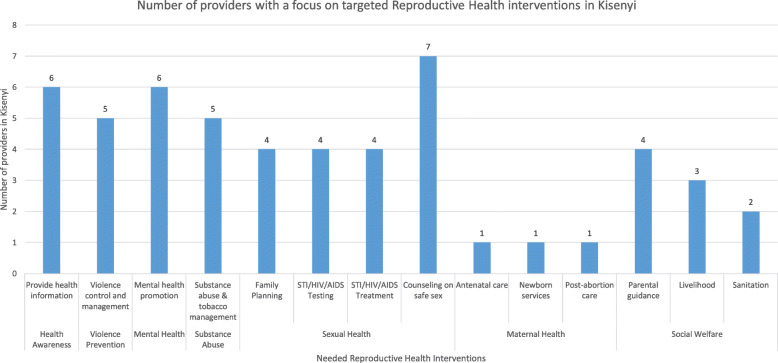
Scope of reproductive health interventions implemented by providers in Kisenyi slum

Figure 1 shows the details of the various RH interventions provided and the number of providers availing each intervention in the respective themes.

#### Design features of the available RH interventions in Kisenyi slum

**Fig. 2:**
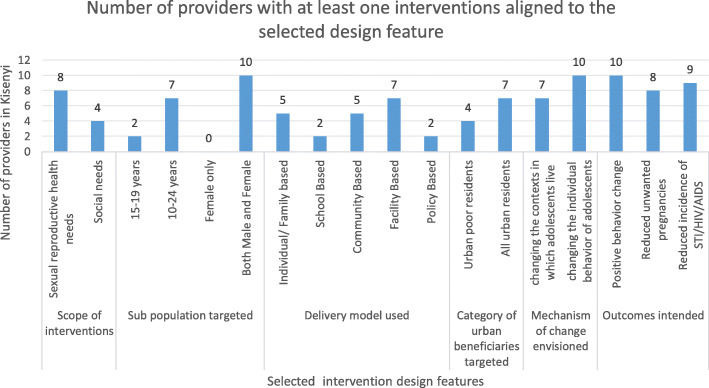
Design features of the available RH interventions in Kisenyi slum

Across the main design features of inquiry described in Table 2 (methods), most interventions by providers focused on sexual health; targeted young people in the age category of 10-24 years; were delivered using a facility based model; targeted all urban beneficiaries – not specific to the urban poor and envisioned individual behavior change as the mechanism of change. Figure 2 summarizes the design and implementation features of the interventions provided in Kisenyi slum. Most outcomes of interventions were related to reducing unwanted pregnancy and lowering the incidence of HIV/AIDS.

The KIs highlighted that the design and implementation of their interventions depended on their understanding of the need and their capacity to provide the respective intervention.

*“We realized that it is important that we separate the adolescents’ services. Their services are considered to be unique and so we try and do that* (provider 3).

*“The adolescents’ antenatal services are not mixed with adults. Their antenatal service is separate …*. B*ut when it comes to delivery … we have the same delivery ward. We have not been able to separate their maternity services* (provider 1).

Most providers organized programs to target young people aged 10-24 years as a whole and sometimes they did not offer the full range of services that the sub category of 15-19 years would need. Most interventions were provided at the health facility or implementing institution’s offices.

*“Our program plans for the youth who are 10-24years and we do link them to health facilities of their choice. We mostly provide preventive commodities like condoms, information and education on condoms” (*Provider 5).

*“For modern contraceptives, we provide information … we do not provide family planning pills in the communities, we can only do referrals to a health facility*” (provider 8).

All providers designed their interventions with the aim of influencing the adolescents’ individual behaviour as the key mechanism of change. Only one provider indicated that they are involved in advocacy work to influence policy that can change the context within which the adolescents live (as a mechanism of change).

“*First of all, I make them my friends, then I get time to interact with them, I go and sit where they sit even when they are in bars, wherever they are. Even for sex workers we interact with them, we make them our friends and then we talk to them and they can accept our services and they change”* (Provider 9).

“*We are so much into advocacy for policy change. For now, we have this policy of comprehensive sexuality education which we are advocating for because we are denied a chance of meeting young people in schools and sharing with them about the problems they are facing. So we are really addressing this policy so much”* (Provider 2).

#### Extent of fit between intervention design features and priority reproductive health needs of the adolescents

**Fig. 3:**
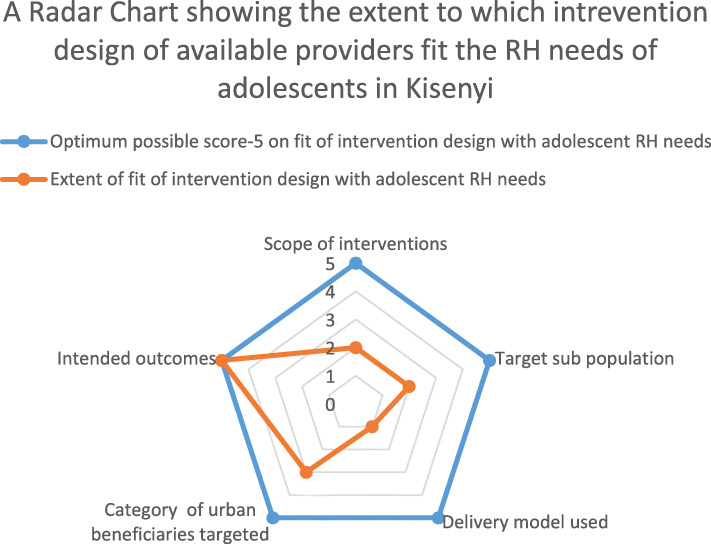
Radar chart of the extent of fit of intervention design features and the adolescent priority RH needs

When the adolescents were asked to score the extent to which the selected design features of the interventions provided in Kisenyi slum fit their priority RH needs, the descriptive analysis showed that generally most interventions were implemented with designs that did not fit the adolescent RH needs in this urban poor context. Figure 3 shows a Radar chart of the extent to which the design of the provided interventions fit the RH needs of the adolescents. For example, the scope of majority interventions was only in moderate fit with the adolescent needs. The adolescents re-iterated that while interventions or service providers comprehensively targeted sexual health, there was a limited focus on social needs like livelihood and sanitation.

*… okay, when you go at the health facility, the health workers are so caring and they give you what came for. But we also need employment. We need employment like teaching girls how to make necklaces and targeting sanitation by giving girls pads …*” *(*Female participant, validation meeting 1).

*“… The vocational institute target only males and even then, you have to know someone there in order to be admitted. One has to also pay registration fees”* (Male participant, validation meeting 2).

Majority of interventions targeted the age group 10-24 years which was in moderate fit with the adolescent needs. The adolescents said that although most interventions targeted the population aged 10-24 years within which they belonged, they preferred interventions that specifically target their age group of 15-19 years for individual beneficiary.

“*They should separate us from the children. We can be seated at the health facility and the health worker starts teaching us things that concern us the adolescents yet young children (10-14years) are also around* …” (Female participant, validation meeting).

The delivery model used by majority of interventions was less fit with the adolescent RH needs. The adolescents were not satisfied with the fact that most interventions that were health facility based. And the few community-based interventions were considered irregular and not holistic. The adolescents preferred regular holistic community outreaches. During the validation meeting, the adolescents specially rated community providers (community health workers, peer educators and community leaders) considerably high in meeting their needs because they were largely working with a community based model. However, they were not happy with the commonly used facility based model and the fact that they had to always be referred from the community to the facility to receive the actual service or commodity needed.

“...*but the providers only come to the community once in a while...you never know when they will come and they only come to offer HIV/AIDS services, yet some of us have other problems”* (Female participant, validation meeting2).

*“Give the health facility zero score because they delay us. You can go there (at the health facility) like at 8am hoping to come back early so that you can go to work but, you end up leaving late even without medicine” (*Male participant, validation meeting1).

*“The VHT members (community health workers) are always available 24/7. The VHT member comes for us from our homes and tells us to go for the services during the community outreaches” (*Male participant, validation meeting1).

Most interventions targeted all urban beneficiaries and not just the urban poor. This was considered to be only in good fit with the adolescent RH needs. During the score of this design feature, the participants insisted that the interventions in Kisenyi slum target both the poor and rich and the poor adolescents sometime end up not accessing what is meant for them. However, some voices (especially among the females) indicated that having interventions that can be accessed by both the rich and poor was good because this protected the adolescents from being discriminated as “slum dwellers”.

*“They don’t discriminate. They treat all of us the same regardless of how you are dressed or if your rich or poor”* (Female participant, validation meeting).

*“Services should be free especially for us from Kisenyi slum … ..But the providers ask us for national identity cards and money when accessing services!”* (Male participant, validation meeting).

The intended outcomes of interest for majority of interventions (reduced unwanted pregnancy and reduced incidence of HIV/AIDS) were in exceptional fit with the adolescent RH needs. The adolescents scored the outcome design highly saying that the intended outcomes aligned perfectly with the adolescents’ aspirations since them too were aspiring to prevent pregnancy and remain free from HIV.

## Discussion

Our study showed that generally, most interventions in Kisenyi slum were implemented with designs that did not fit the adolescent RH needs. The available RH interventions focused on meeting sexual RH needs but not addressing social needs like livelihood and sanitation which the adolescents identified as equally important. While the providers designed intervention to target 10–24 year olds, the adolescents preferred to have interventions that specifically targeted their age group of 15–19 years. Most interventions were facility-based while, the Adolescents desired regular holistic community based outreaches.

### Interventions in Kisenyi perceived not to meet the RH needs of the adolescents in this urban poor setting

The interventions that providers were implementing in Kisenyi were considered not to meet all the adolescents’ RH needs. The adolescents’ needs ranged from need for specific services and commodities to need of services delivery models that matched the adolescents’ demographic profile and living conditions. The adolescents in Kisenyi and specifically the ones we had discussions with had unique characteristics that compounded their vulnerability e.g. they live without parents or caretakers, some head homes, many are single parents, dropped out of school and some have never gone to school, are exposed to drugs and alcohol, they are poor and lack employment yet they have to provide for themselves. They also have poor standards of living especially housing and sanitation, lack health information hence not empowered to negotiate safe sex. All these contextual factors [13] have to be considered by urban authorities and implementers who plan to reach these adolescents with interventions/ services.

It is important to clearly map out needs of adolescents in different contexts [36]. The urban poor context requires that the scope of RH interventions for adolescents is expanded to accommodate context specific needs (e.g. livelihood and community based delivery models) which in themselves if not provided can reduce accessibility (physical, financial and acceptability) to the available RH interventions [37, 38]. This can improve the effectives of health programs which are greatly needed by the adolescents [6, 39].

### Reproductive health needs are broader than sexual health services and information

Our study shows that RH needs encompasses aspects other than sexual RH services and information. The adolescents identified another dimension of social welfare as an important RH need for them. Social needs include livelihood, housing, sanitation and parental guidance. Although the adolescents considered social welfare interventions to be important for RH, very few providers were implementing interventions to meet social needs hence the adolescent felt that by design, the scope of the available interventions did not meet their needs. In the context of the urban poor, RH intervention implementers need to adjust the design of their interventions to also tackle social needs in order for the adolescents to have a holistic RH [24].

These social needs especially livelihood modify the context in which the adolescents live in order to remove vulnerability [21]. Poorer adolescents face significant barriers in realizing their reproductive health and rights [40] and WHO guidelines stress the role of educational and employment opportunities in helping adolescents make a place for themselves in the world. It is recognized that in many places, adolescents have limited opportunities to grow and develop to their full potential, and that poverty and insecurity among other issues, increase their vulnerability to health and social problems [19]. The female FGD participants had a perception that poor sanitation resulting from poor toilet facilities and trenches in their community affected their RH causing urinary tract infections and poor menstrual hygiene an issue that had been raised in other studies [41]. This demonstrates the direct linkage of social basic needs with reproductive health.

### Design of current RH programs not packaged to meet RH needs of adolescents in urban poor contexts

The sexual RH needs that the adolescents prioritized in our study did not differ from other studies [7, 42]. However, the adolescents made an emphasis on quality and adequacy (e.g. quality family planning and quality HIV/AIDS testing and treatment). This seemed to indicate that the quality of sexual RH services and information was lacking. Quality issues have also been documented elsewhere such as lack of “safe spaces” for adolescents and having providers whose demographics does not match that of the adolescents’ profile [43]. We hypothesize that the concerns around quality and adequacy may be influenced by how the interventions are designed, packaged and delivered. For example, most family planning interventions were delivered using a facility based model yet the adolescents preferred community outreaches. This mismatch in delivery model preferred and the delivery model used can influence the extent to which the intervention fit the RH needs of the adolescents. The Uganda adolescent RH service standards and the WHO global standards framework provide guidelines that can be used to deliver components (information, service and products) of adolescent friendly health services [17, 24].

When planning and providing RH services for adolescents, designers and implementers of RH interventions need to be aware of the adolescents’ preferred design characteristics for interventions. This is because, access improves as health care services become better aligned with clients’ needs and resources [12]. In our study, we noted that the design features of most interest were: scope of the interventions (adolescents needed interventions that meet both sexual and social welfare needs); sub population targeted (adolescents preferred interventions that specifically targeted their age group of 15-19 years); and delivery model (adolescents preferred regular holistic all round community outreaches). Related considerations have been articulated in the Uganda adolescent RH service standards that mentions *“appropriate; relevant services provided as per specific needs and circumstances of adolescents based on age, sex, marital status and socio-economic situation* [17]*.* In Kisenyi, the adolescents actually rated the community health workers, peer educators and community leaders considerably high in meeting their needs because they are largely working with a community based model. We also note the need to ensure that when a community based model is adopted, issues of stigma are managed. In our study, we noted that the adolescents’ aspirations aligned well with the interventions’ envisioned outcomes (reduced HIV/AIDS incidence and reduced unwanted pregnancies). Such an intervention design characteristic that matched with the adolescents’ needs should be maintained by implementers.

Providers are encouraged to have a holistic scope of services and to package medical and social interventions in order to meet the adolescent RH needs. An example of such innovative health programming is the multi-sectoral DREAMS core package of interventions [44] that goes beyond the health sector to “address the structural drivers that directly and indirectly increase girls’ HIV risk, including poverty, gender inequality, sexual violence, and a lack of education”. In a complex setting like the urban poor, implementing adolescent RH interventions also requires complex design where a number of interventions are packaged and delivered both independently and interdependently for example investing in livelihood interventions for adolescent girls in order to empower them and improve their negotiation power in relationships which ultimately improves their reproductive health. It is therefore necessary to understand whether “implementation design is adequate” for the given context.

### Implications for public health programming

The interaction between urban health and RH is a growing area of focus given the increasing population growth rate and increase in informal settlements in Low and Middle-Income Countries (LMICs) like Uganda [45, 46]. When developing and implementing interventions, providers need to take into account that while many adolescents and young people share common characteristics, their needs vary by age, sex, educational status, marital status and urban residence among other characteristics [17]. Implementers need to appreciate the need to work in different ways with different groups in different contexts, in complementary ways and abandon approaches that are wasteful and ineffective [47]. It is hoped that designers and implementers can increasingly have an understanding of intervention characteristics that fit better the needs of the urban poor especially for adolescent RH. This aligns with the Universal Health Coverage (UHC) agenda that Uganda and many other countries has committed to in the efforts towards sustainable development and poverty reduction, and ultimately reducing social inequities.

### Study limitations

This study did not stratify the validation meetings according to in school and out of school hence missed out on some detailed insights on how best interventions should be delivered for the in school and out of school adolescents. Our study used the adolescents’ perspective to assess the extent to which the available RH interventions and the selected design features fit their RH needs. We did not probe the service provider’s perspectives on how they would like RH interventions to be designed and delivered. Details on the underlying mechanisms of change and program assumptions for the various interventions were not evaluated by the adolescents which fell short of complete realistic evaluation [32]. The eligible adolescents were purposively identified by the VHTs who we believed knew best the adolescents who live in Kisenyi and could consent them. However, we note that this may have introduced bias since it was at the discretion of the VHT.

## Conclusions

There was a perceived mismatch between the RH needs of the adolescents and the RH interventions provided in Kisenyi slum. The scope of interventions was mainly limited to tackle sexual RH like providing family planning services and HIV/AIDS testing and treatment, yet the RH need of the adolescents also included social needs like livelihood and sanitation. While interventions were mainly facility-based, the Adolescents desired regular holistic community based outreach delivery models. Our study demonstrates a first step in having a structured movement from the identification of needs to adaption of interventions to fit the context of urban poor [48] in terms of design and implementation.

### Recommendations

Designers and implementers of RH interventions need to be aware of the adolescents’ preferred design characteristics for interventions. Our findings support better alignment of programs designs to the needs of adolescents by improving 1) the integration of RH needs with livelihood support through service providers’ purposeful investment in livelihood opportunities as an RH intervention for adolescents; 2) community-based models and outreach programs and less facility-based approaches while managing issues of stigma; 3) designing ways to improve access by the urban poor communities by removing barriers to access such as fees and long waiting time as examples. Programs that are effective in reducing pregnancy and HIV infection are highly preferred alongside improving livelihoods of adolescents.

## Data Availability

The datasets used and/or analyzed during for our study are available from the corresponding author upon reasonable request.

## Abbreviations

AIDS: Acquired Immunodeficiency Syndrome
FGDs: Focus Group Discussions
HCIV: Health Center IV
HDREC: Higher Degrees Research and Ethics Committee
HIV: Human Immunodeficiency Virus
KIIs: Key Informant Interviews
LMICs: Low and Middle Income Countries
MakSPH: Makerere University School of Public Health
NGO: Non-Government Organization
Ref: Reference
RH: Reproductive Health
SDG: Sustainable Development Goals
SRHR: Sexual Reproductive Health and Rights
SRHS: Sexual Reproductive Health Service
STls: Sexually Transmitted Infections
UHC: Universal Health Coverage (UHC)
VHT: Village Health Team
WHO: World Health Organization
